# Roma Housing and Eating in 1775 and 2013: A Comparison

**DOI:** 10.3390/ijerph15040588

**Published:** 2018-03-25

**Authors:** Michal Kozubik, Jitse P. van Dijk, Barbora Odraskova

**Affiliations:** 1 Department of Social Work and Social Sciences, Faculty of Social Sciences and Health Care, Constantine the Philosopher University in Nitra, 949 74 Nitra, Slovak Republic; mkozubik@ukf.sk; 2 Department of Community & Occupational Medicine, University Medical Centre Groningen, University Groningen, 9713 GZ Groningen, The Netherlands; 3 Olomouc University Society and Health Institute, Palacký University, 771 47 Olomouc, Czech Republic; 4 Department of Social Medicine and Public Health, Faculty of Medicine and Dentistry, Palacký University, 771 47 Olomouc, Czech Republic; 5 Institute of Romany Studies, Faculty of Social Sciences and Health Care, Constantine the Philosopher University in Nitra, 949 74 Nitra, Slovak Republic; barbora.odraskova@ukf.sk

**Keywords:** Roma, housing, eating habits, comparison, 18th century, 21st century, Slovakia

## Abstract

We compared housing and the eating habits of Roma. Contemporary findings (2013) were compared with those from the first monothematic work on Roma (1775), which depicts their housing and eating habits, especially regarding the differences between social classes. Data were obtained from a journal (1775) and from semi-structured interviews (2013) with more than 70 Roma women and men who live in segregated and excluded settlements at the edges of villages or scattered among the majority. Data were collected in two villages and one district town in the Tatra region, where the data from the 1775 measurements originated. We used classical sociological theory to interpret the obtained data. The main findings showed differences between specific social classes then and now regarding housing, as well as the eating habits related to both conditions among the Roma in the Tatra region. The houses of rich Roma families did not differ from the houses of the majority population. The huts of the poorest inhabitants of settlements did not meet any hygiene standards. Typical Roma foods such as *gója* or *marikľa* were the traditional foods of Slovak peasants living in poverty in the country. We concluded that the housing and eating habits of the citizens of poor settlements located in the eastern parts of Slovakia are still similar to those of two centuries ago. The existing social exclusion may be explained partly from this finding.

## 1. Introduction

A general picture of the development of public health and its understanding is known [1,2,3]. However, the more than 200-year development in hard to reach groups is generally not very well known. Roma health has been a neglected topic of research for several decades [4]. In recent years, however, the topic of Roma health has come into the spotlight [5,6,7,8], sometimes showing what was already known and sometimes coming up with new findings. Roma health includes a low level of recognition and understanding of the minority by the majority, a topic which is very difficult to study. We focused on two issues related to health, i.e., the housing and eating habits of the Roma, and studied them from an anthropological and historical perspective. Evidence among these topics in this group is lacking.

In the past, Roma eating patterns were related to their way of life. Most Roma worked in craftsmanship, agriculture, or as ancillaries in the households of wealthier families. Kacala et al., Kotzmanova, Rimarova and Lovayova [9,10,11] studied nutritional patterns in 300 9–13-year-old Roma children and suggested that the classic Roma diet did not include fruits or vegetables, and that milk and dairy product intake was very low. They evaluated that these were highly unhealthy habits in an ethnically specific population. Hancock [12,13] stated that the majority of the population made a fundamental error in their judgement: Roma culture is often confused with the concept of the culture of poverty [14]. Preparing food among the Roma has in the past always been women’s work [15,16,17]. Current studies, however, have perceived significant changes in the status of men and women in the Roma community, a supposed result of the emancipation of Roma women [18]. The population living in Roma settlements has more frequently reported unhealthy eating habits when compared with the majority of the population. Such habits might contribute to the worsening health status of the Roma population. These differences might be attributable to cultural differences between ethnic as well as socioeconomic groups [19,20].

The housing of the Roma has been discussed by authors in Slovakia and from abroad [21]. Horvathova [22] depicts Roma housing in ancient history, where their dwellings were mostly caravans, marquees, and tents usually made of canvas and constructed in the shape of a cone. It was possible to keep a fire in the front or back part of the tent in such a way that smoke could get out. These Roma housing facilities were replaced with huts after the Second World War [23]. The Košice Governmental Program, declared on 5 April 1945, proclaimed the rejection of discrimination because of racial and religious reasons. Despite that statement, state policy stimulated hidden or open forms of forced assimilation. Law No. 74/1958 “on the permanent settling of nomadic persons” permanently restrained the movement of the traveling portion of the Roma population (Vlachike Roma). In the same year, the Communist Party of Czechoslovakia issued a resolution whose aim was “the consistent assimilation of the Gypsy population” [24]. The Roma were forced to live in blocks of flats, in housing estates or at the edge of municipalities, i.e., in accommodation provided by the municipalities. Since then, Roma ethnic groups in Slovakia have practically lived in segregated settlements, separated communities, or scattered in towns. According to the most recent report from the European Commission [25], the lives of the Roma have improved in the scope of housing and health care in EU countries, however, 80% of Roma are still at risk of poverty. The EU health strategy “Together for Health” (supporting the overall Europe 2020 strategy) aims to turn the EU into a smart, sustainable, and inclusive economy promoting growth for all, including the Roma. Current challenges are to promote health, prevent diseases, and foster supportive environments for healthy lifestyles [26,27,28]. Current studies have discussed a new phenomenon of ghettoization where hygienic conditions in the scope of Roma housing in Western European metropoles have been worsening [29,30,31,32]. 

The areas of housing and health are closely related. Both of them are perceived as barriers to immunization in the Roma [33] and result in multiple health complications [34]. Life expectancy (LE) is considered to be a basic indicator of a population’s health status. The European Commission estimates that in Slovakia, LE is 55.3 years in Roma men and 59.5 years in Roma women [35]. According to Infostat in the Slovak Republic, LE is 64.4 years in men and 71.6 years in women [36]. Furthermore, the worst health outcomes are shown for physically segregated communities, home to approximately 40% of the 450,000 Slovak Roma [37]. For these places, many studies claim worse self-rated health [37,38,39]. Demographic projections have reported higher mortality rates and a shorter LE among the Roma [37,40], and clinical studies have shown a significantly greater communicable and non-communicable disease burden across the life-course. These poor health outcomes seem to result from adverse circumstances in segregated communities [41,42,43,44,45,46]. 

Thus far, a comparison of contemporary data with information from the past [47,48] on eating habits and housing (focusing on segregated settlements, separated communities, and Roma living in towns) has been lacking as a part of the history of public health [1,2]. In this study, we compared the eating habits and housing of Roma in 1775 and in 2013. Our research objective was to analyze the content of the first work, which was issued between 1775–1776 in a Vienna weekly [47,48], and to seek parallels between the past and present situations in the context of the processes of change to the Roma living in the region below the Tatras.

## 2. Methods

We conducted our field research in the same area that Augustini wrote about in the 18th century. Furthermore, we worked with the settled Roma in the Poprad district, as it is known that the Roma inhabitants have not moved from this region [24]. We focused only on those sociocultural norms related to eating and housing, according to a reductionist definition of culture [49,50,51] in the context of health. A thorough anamnesis of the semi-structured interviews preceded the ethnographic field investigation. 

### 2.1. Samples

The Atlas of Roma Communities [52] states that there are 402,840 Roma living in Slovakia. This is 7.5% of the total population, far more than the 2% (105,700) suggested by the most recent Census 2011 [53]. In our study, almost all Roma openly reported themselves to be Roma (90%) and all of them did not have any problem talking about cultural specifics. 

**Figure 1:**
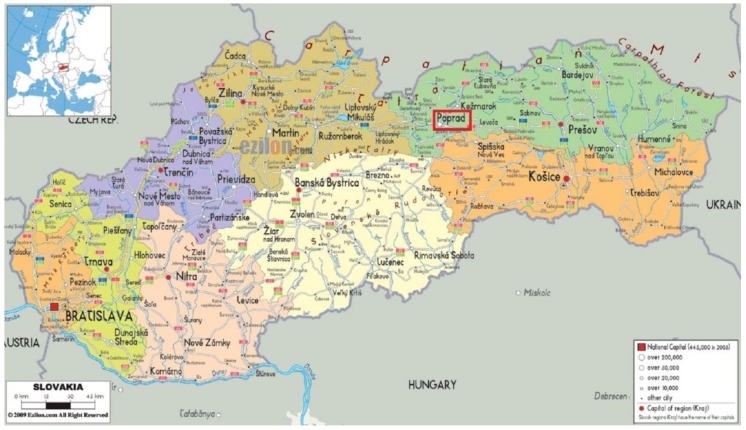
Slovakia, showing the city of Poprad (red rectangle) Source: Slovakia-Central Europe (2016) [56].

**Figure 2:**
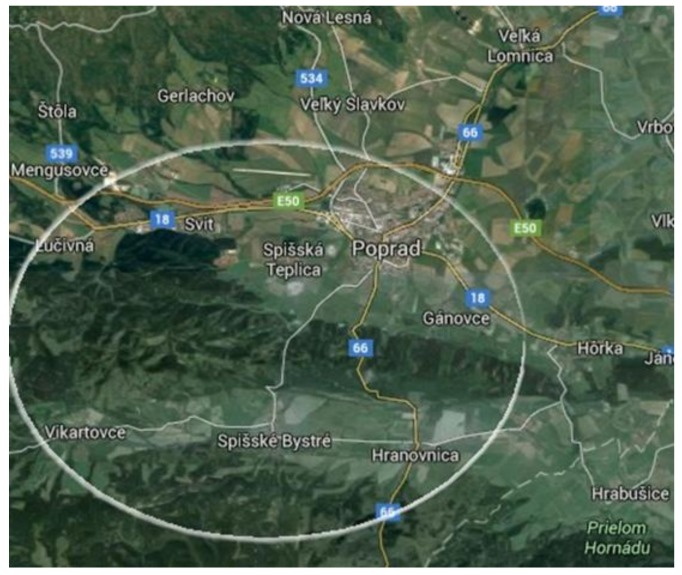
Data collection sites (Poprad, Spišská Teplica, Hranovnica, Spišské Bystré, Vikartovce). Source: Authors.

In line with Radicova [54], we described the three groups as dwellers in integrated or separated communities, or segregated settlements. Regarding ethnicity, we worked with the *Rumungre* Roma living in the east of Slovakia [55]. We used random sampling with no defined criteria. In our field research conducted in 2012–2013, we interviewed more than 50 people, conducted two focus groups, made more than 1700 min of recordings, and took more than 250 photographs. At the beginning of the research, local intermediaries introduced us to the families who provided us with a place to stay. The interviews on nutrition patterns were conducted in family settings. Then, we visited randomly chosen houses and huts. Some people even sought us out and wanted to share their everyday reality. The semi-structured observations and interviews took place in the natural environment of the dwellers in the three types of settlements. The data were collected in three localities of the Poprad district: a segregated settlement, a separated concentration, and the town itself, where the Roma live integrated among the majority of the population (Figure 1 and Figure 2).

### 2.2. Data Collection

Data were collected through ethnographic field research. This focused on a detailed, in-depth analysis of the sociocultural norms and ideas of the Roma on eating and housing as dwellers in separated communities and segregated settlements. Ethnographic research should include a stay in the field [57,58], which is an indispensable activity leading to the detection and recognition of social practices. We stayed in the three mentioned contexts: the town part, the separated community, and the segregated settlement. The field work was conducted in the summer months of 2012 and 2013. Developing trust with the settlement dwellers happened step by step. Therefore, in 2012, field work included all-day visits to the segregated location. Only in 2013 did we decide to stay directly in the respondents’ places. We were able to collect and compare individual sociocultural norms and ideas in the selected localities with those stated by Augustini more than 200 years ago [59]. The ideas and opinions of a significant author of classical sociological theory (Weber [60,61,62,63]) inspired us in our data interpretation. Weber emphasized that each study is a motivated conduct, which is guided by the researcher’s values or “value ideas”. He thus insisted on the fact that this justified a link (*Wertbeziehung*) to the researcher’s values and that consequently their study should be removed from their values. There should be no mixing of the researcher’s value judgement (*Werturteil*) and their assessment of the facts, which the researcher must conclude on the basis of the data obtained. The data were collected in such a way that their relative assessment earns the consent of the other actors, who may not share the attitudes and values of the researcher.

### 2.3. Analyses and Reporting

Reporting in this paper was based on the two main variables: eating and housing. For both of these variables, the situation in 1775 from Augustini’s publication [47] is described first, followed by the 2013 situation based on our own experience. Augustini’s work was published in 1775 and 1776. One of the most important challenges from that period in the context of eating and housing was the differentiation between the studied community and the generalization of all of them. He critically explored all contemporary and previous literature, hand-written materials, and other historical sources. He summed up data from the literature and sources through the depiction of the current state of the Roma based on his own and his contemporaries’ observations. Furthermore, he emphasized that the information came from his own experience and their statements [47].

We used comparable methods. The analysis of Augustini’s work was followed by a detailed description of the habits and traditions in the past and a comparison with the present-day life of the Roma. We conducted the research in the same geographic area as Augustini did in the 18th century. The general scheme of our ethnographic analysis was clearly structured and consisted of setting the research objectives and characteristics of the culture, then the analysis of the topics and their interpretations: Topic 1 (eating)—interpretation 1; and Topic 2 (housing)—interpretation 2. Data on contacts, informants, transcription, family trees, places, and diaries were used to interpret the topics. Data were analyzed using the method of thick description [50,51]. The aim of such description was to come to conclusions from the minutes, while remaining closely related to the data with precisely-defined complex specifications. This description presented a cultural reality as an interpretatively open and complex structured and layered system of meanings, where each area of human behavior or statements about a certain human phenomenon could be extended into new contexts and reinterpreted by any other person entering a problem or situation that was the subject of interest. This is how we derived our conclusions from the data, enabling us to obtain conclusions with social implications. 

### 2.4. Research Ethics

In our manuscript, neither animals nor plants were studied. Human beings from 1775 were not studied by us, and human beings from 2013 were studied in line with the Helsinki Declaration. All of the participants agreed to their participation in this study. Their informed consents were obtained and archived through audio recordings.

## 3. Results

Our results are presented through the direct statements of the interviewees. The information was not modified. We considered it very important that the informants were able to express themselves in their own words, by literal depictions—translated by the authors—of their everyday reality. The text below depicts the scope of eating habits and housing in the two compared historical periods.

### 3.1. Roma Food and Eating 1775

Augustini’s contribution was not only a depiction of the living conditions of the Roma in Hungary in the 18th century, but also broke down many of the stereotypes about them and the criticism of contemporary reports about this ethnic minority. Roma who earned a living by their own hands were evaluated as being significantly different from the others: “sometimes they eat bread, too” [47], but they did not bake it themselves, because they did not have suitable conditions in their dwellings to do so. If the wealthier families could not procure meat, they ate mostly flour dishes, which they prepared in the embers directly in the hearth. Contemporary texts unambiguously show the diversity of the Roma community, particularly specific clans in wealth or poverty. This difference was reflected in the way of obtaining food and the quality of eating. While the rich families normally ate bread and meat, the poor had to settle for carrion. Roma did not eat horses as they were important for their life (travelling), and they used only the skin of dead animals for fur. 

Augustini’s statements could be seen as stereotypes and prejudices, however, they have to be understood in the context of the period in which they were found. Nowadays, Augustini’s attitudes appear as scathing criticism. His work as a whole, however, is the greatest contemporary defense of the Roma [47]. 

### 3.2. Roma Food and Eating Now

In the present-day Roma community, food is a measure of wealth and poverty. It is also used to express love to children, guests, family, or unknown wayfarers. The success of the breadwinner is measured by the amount of food he is able to procure: “*I have earned and brought five full bags!*” (36 years old, male). For the father, children, and their mother, the nicest periods are when they are provided with everything they need for their life. However, it is not unusual for a Roma to buy tens of kilograms of flour on the days when receiving welfare benefit. After several days, when the savings have been spent, the family mixes flour with water and makes the most modest food, the so-called marikľa. Marikľa is thin bread, made of flour, water, soda or a bit of baking powder and salt, and it is formed and put on the oven, normally from one side. “*And then when children come to school in the morning and we ask them what they have eaten, they say marikľa*” (45 years old, female). 

For Roma, the best food is *riska* (cutlet, schnitzel) with potatoes. *Fľaky* (elsewhere also called *gója*) is considered a Roma specialty and their typical food [59,64,65]. *Fľaky* consists of a skin filled with grated potatoes, pieces of meat, flour, eggs, and spices (salt and pepper). The skin is filled with this mixture, and then it is boiled over the fire. It is a really old recipe, the food of people who work hard in the fields every day and is ultimately the food of poverty. A 77-year-old Slovak informant stated: “*we have prepared flaky since I was a child. It was the food of the poor*”. Most of the majority of the population stopped making and eating “*fľaky*”, but the Roma continued to make it. The legend about famous *fľaky* might have appeared because of this. The poor Roma neither stored food nor had food supplies: “*You have to eat it; otherwise we will throw everything away! Our habit is to throw away everything that has not been eaten*” (31 years old, female). Roma did not have refrigerators in the huts as a consequence of not having electricity. Sometimes a car battery is used for the TV or a bulb. There are some refrigerators in the settlements, but they are used for storing food rather than keeping it cold. 

Both the eating habits and consumed foods are astoundingly similar in both compared periods. Unambiguously, they represent the life strategies of people living in poverty and are not a traditional characteristic of the Roma culture. One of its most significant features is hospitality, not food itself. The above-mentioned *gója* is an old traditional food of the peasants and poor Slovak farmers. *Marikľa* (flour mixed with water baked on a fire) is a symbol of poverty of the poorest families in the community. Furthermore, nowadays, Roma are used to consuming meat from dead animals, dogs, and even horses [66]. The foods of the wealthiest Roma families do not differ from common Slovak foods at all. Family parties or ceremonies, however, are significantly richer in food choice, in comparison with the majority.

### 3.3. Roma Houses and Housing 1775

Roma, in the time that Augustini described them, were either settled or nomadic [47]. Roma in Hungary and Transylvania settled solely in the places that were selected for them to live in. Augustini [47] mentions the towns of Sibiu (now Romania), Debrecen (still Hungary), Bystrica, Prešov, and Košice (now Slovakia). These groups of Roma mostly lived a settled way of life. The nomadic groups of the period around 1775 included the Moldavian (*Lach*), German-speaking Roma, and the *Lyngurars*. Nomadic Roma lived in tents. They liked those dwellings the most as they enabled them to move from one place to another very quickly. They travelled often, but never far away. Usually they stayed near the county where they were born. In the cold months, they built winter dwellings, which were spaces dug in the ground, supported by logs, and lined with straw. The entrance faced the south or the east. When the weather became warmer, they demolished these dwellings and lived in tents again. 

Roma living in towns and their vicinities were richer and perceived themselves as better and more distinguished. A nomadic way of life was typical for poorer Roma communities. In the studied region of Poprad, a mountainous area, before 1800, Roma lived in specially-dug dwellings in the ground in winter. Augustini described their construction in detail. In summer, they travelled with horses around the area and stayed in tents.

### 3.4. Roma Houses and Housing Now

The Slovak literature depicting the housing of the Roma differentiates between integrated and separated communities, and segregated settlements [54,65]. This differentiation indicates the heterogeneity of the Roma community in general, not only regarding housing. Most of the Roma living “integrated” live in blocks of flats and family houses in parts of a town, in our case Poprad. Roma and poor families of the non-Roma majority live next to each other. The living conditions are rough. There are often multi-member families squeezed into small “flat units”. In villages like Kravany, the Roma live concentrated at the edge of the municipality in a separate settlement. In the separated and segregated localities, like Hranovnica, houses for three Roma social classes exist. Members of the high class live in common brick houses, the middle class also live in brick houses, but they are more neglected and crowded while the lowest class live in modest wooden huts. This is an image of poverty in the 21st century. Many brick houses, wooden huts, and sheds, of considerably different quality, can be seen in the settlements. 

In the present day, Roma do not set up any tents or winter dwellings. This is the most significant difference in comparison with 1775. Like eating, housing is also a status issue. Colors bordering on kitsch are popular, together with garden statues or vastly decorated facades. This is mostly a demonstration of higher social status in the settlement or separated locality. Despite the fact that only a few houses are connected to the municipal sewer system, all of them have a toilet and bathroom connected to the municipal water supply. The wooden huts, however, do not have these facilities. Thus, families with children living in such accommodation face a great risk of many diseases. The ghettos in towns are characterized by the monotony and homogeneity of concentrated poverty, which exists regardless of ethnicity (common social housing for both the Roma and the Slovaks).

## 4. Discussion

Our objective was to compare the food and housing of the Roma community of Eastern Slovakia depicted in *Zigeuner in Ungarn* (1775) with the present (2013). We found that the food and eating habits have not greatly changed over the centuries. An exception is the consumption of horse-meat, which was taboo in the past. The poor consumed—and still consume—food of low quality, ate irregularly, and often went hungry. The Roma food presented as traditional was the food of poor Slovaks in the past. The eating habits and food of the Roma did not differ significantly from the eating habits of the non-Roma Slovaks. Furthermore, we found that Augustini’s detailed description of the construction of winter dwellings and tents was important historical material that, however, did not correspond with the housing situation today. Like eating, housing is also a demonstration of social status in the Roma community. In the rural settlements, simple wooden huts can be found in segregated settlements and brick houses can be found in separated communities, i.e., parts of villages that are mostly inhabited by Roma families. The urban living quarters and housing can be considered as town ghettos that are fully occupied by poor citizens with no cultural differences.

### 4.1. Eating

We found no differences in the eating pattern over more than two hundred years, except regarding the consumption of horse meat. Augustini [47] stated that the Roma consumed carrion because of their opinion that the “*meat of an animal killed by God must be better than meat of animal killed by the hand of a man*”. Furthermore, he stated that horse meat was not eaten by the Roma because of a certain reverence for horses. This reverence, however, has disappeared in the third millennium. There was a case in Richnava where the local Roma stole and ate a horse [66]. In the present environment of the settlements below the Tatras, we found that only dog fat was used for the treatment of airways and lungs and was an expensive “export commodity”. It was used as an ointment but was sometimes also eaten in small amounts as grease. Eating habits are an important link between the food of poverty and traditional Roma foods (particularly *gója* and *fľaky* or *marikľa*). Our ancestors knew these foods and consumed them normally. In rural areas, they are known and popular among older people. The eating habits and food of the Roma in the time when Augustini lived and the present have not been previously compared by any authors. The life strategies of the poor Roma focus mainly on the present and bare survival, which is typical behavior in socially excluded communities [13,14].

### 4.2. Housing

The biggest change in housing is the change in the nomadic way of life. The Roma have stopped using tents and do not build provisional winter dwellings as described in detail in Augustini’s work. The Roma in Slovakia no longer have any reason to live in tents or to build winter dwellings. In our opinion, the causes of changes in building and the character of dwellings were a result of the events from the above-mentioned 1950s, when Law No. 74/1958 [67] on the permanent settling of nomadic persons restrained the movement of the Roma. Today’s social stratification can be seen in great detail. At present, the houses and huts mirror the social stratification of each Roma community (brick houses for the rich, wooden huts for the poor). Poor Slovak groups, including the homeless as well as the poor Roma, concentrate in emergency housing facilities in areas defined by the authorities of villages and towns (Poprad). The local areas in the neighborhood of villages (e.g., Kravany) consist particularly of brick houses. The inhabitants of segregated settlements mainly live in wooden huts that do not meet any hygienic standards (e.g., Hranovnica).

### 4.3. Strengths and Limitations

We have compared the previously unanalyzed scope of eating habits and housing of a minority group from a historical perspective. This study was conducted exactly in the environment in the north-east of Slovakia where Augustini lived and worked in the 18th century. The study was based on long-term field data collection that analyzed many other habits and traditions, in addition to those presented here, and formed part of a longer study report [47,48]. A specifically-defined geographic area was analyzed, and the idiographic approach describing the processes in Roma in this area was used. Therefore, the results cannot be generalized for the whole Roma population in Slovakia. Results came from the specific minority group, the so-called *Rumungre Roma*, living in the north-east part of Slovak Republic.

### 4.4. Recommendations

The problems of reproduced poverty accumulated in segregated Roma settlements have not disappeared even after two centuries. Many attempts to improve food aid for poor Roma communities have failed [68]. The most highly acknowledged project funded by the EU is the self-help Roma housing development, conducted in Spišský Hrhov (30 km from the studied area) [69]. It is a positive example of good communal vision and policy. To combat poverty in the present day, municipalities should establish community centers and provide social work in the field, which is what most of them are doing. The work of both of these types of institutions (community centers and field social work) has been conducted unsystematically: one project is merely replaced with another. The challenge for politicians should be the creation of a social security system that would provide systematic aid and thus meet people’s basic needs, including housing and eating. The objectives of any social system should therefore include attempts to create an effective combined system that provides aid for persons living in poverty. 

Despite the high-quality study reports and analyses mentioned in the introduction to this paper [4,5,8], in Slovakia there has been a lack of research studies that deal with the eating habits of the poor in the settlements. Future research projects, therefore, including multi-disciplinary teams consisting of social scientists and health professionals, should focus on analyzing needs in the areas of eating and housing in the settlements. In addition, the above-mentioned political measures should be thoroughly re-evaluated by multi-disciplinary academic teams. The resulting data might make food and housing aid more effective, since it has so far been unsystematic in Slovakia [68]. 

## 5. Conclusions

We found that the food and eating habits of the Roma have not greatly changed over the centuries, however, housing has changed. The traditional Roma food *gója* was the food of Slovak peasants in the past. It was considered to be a meal for poor people. Nowadays, with this exception, Slovak and Roma food does not significantly differ. The settled Roma lived in towns and their vicinities. They perceived themselves as better, richer, and more distinguished. The poor families led a nomadic way of life. In the summer they lived in tents, and in the winter, they dug special dwellings in the ground. Today, the Roma do not lead a nomadic way of life. Poor Roma live in wooden huts with no connection to electricity and water. 

The challenge for politicians should be the creation of a social security system that would provide systematic aid, thus meeting people’s basic needs, including housing and eating. The objectives of any social system should therefore include attempts to create an effective combined system that provides aid for persons living in poverty. Such political measures should be evaluated by multi-disciplinary academic teams to make food and housing policies more effective.
